# Long-term performance of oxidized zirconium on conventional and highly cross-linked polyethylene in total hip arthroplasty

**DOI:** 10.1051/sicotj/2020010

**Published:** 2020-05-07

**Authors:** Alberto V. Carli, Anay R. Patel, Michael B. Cross, David J. Mayman, Kaitlin M. Carroll, Paul M. Pellicci, Seth A. Jerabek

**Affiliations:** 1 Hospital for Special Surgery 535 E 70th St. New York 10021 NY USA; 2 Fondren Orthopedic Group 7401 Main St. Houston 77030 TX USA

**Keywords:** Oxidized zirconium, Highly cross-linked polyethylene, Total hip arthroplasty, Long-term outcomes, Osteolysis

## Abstract

*Introduction*: Polyethylene wear and subsequent osteolysis remain obstacles to the long-term survivorship of total hip arthroplasty (THA). Highly cross-linked polyethylene (XLPE) with radical quenching represents a massive leap forward with dramatically improved wear rates compared to ultra-high molecular weight polyethylene (UHMWPE). In this study we evaluate the wear of UHMWPE and XLPE coupled with oxidized zirconium (OxZr) femoral heads. *Methods*: A longitudinal, retrospective analysis was performed identifying consecutive patients who received a 28-mm OxZr-on-polyethylene primary THA from 2003 to 2004 by a single, high-volume arthroplasty surgeon. Patients were divided into two groups: those that received (1) UHMWPE liner and (2) a highly XLPE liner. Patients were included if clinical follow-up was complete to 2014 or later. Radiographic analysis was performed by two blinded observers. Measures included cup position, annual linear wear rate, and presence of osteolysis. Pairwise comparisons, correlations, and inter-rater reliability were calculated. *Results*: Eighty patients were in the UHMWPE group with an average follow-up of 10 ± 1.23 years and 88 patients in the XLPE group with an average of 10 ± 1.03-year follow-up. Average age (68) was similar between groups (*p* = 0.288). Observer reliability was excellent for cup abduction (ICC = 0.940), anteversion (ICC = 0.942), and detection of osteolysis (ICC = 0.811). Annual linear wear rates were significantly higher (*p* = 1 × 10^−19^) with UHMWPE (0.21 ± 0.12 mm/year) compared to XLPE (0.05 ± 0.03 mm/year). Linear wear rate was significantly correlated to decreasing acetabular abduction (*p* = 0.035). Osteolysis was noted only in the UHMWPE group, with 17 patients (21.2%) exhibiting acetabular osteolysis and 37 (46.3%) patients exhibiting femoral osteolysis. *Conclusions*: OxZr coupled with XLPE showed minimal wear and no osteolysis at 10-year follow up. The yearly linear penetration rate is similar to that seen in other studies of XLPE THA. A careful longitudinal follow-up will be required to determine if advanced bearings such as OxZr or ceramic can show improved performance in the second decade of implantation.

## Introduction

Polyethylene wear and subsequent osteolysis remain the most common reason for revision in the medium to long term following total hip arthroplasty (THA) [1, 2]. Particulate debris generated by polyethylene produces an inflammatory tissue response that leads to retroacetabular and femoral osteolysis, with subsequent loosening of these components [3]. Previous investigations have associated a causal link between wear debris and osteolysis [4] and a polyethylene linear wear rate of 0.1 mm/year is widely quoted to be the threshold for which osteolysis occurs [5]. Efforts to improve polyethylene wear resistance resulted first in the production of ultra-high molecular weight polyethylene (UHMWPE) in 1971 [6], with subsequent refinements occurring throughout the 1990s [7, 8]. The second and most recent major effort to improve polyethylene wear resistance was the production of highly cross-linked polyethylene (XLPE), which was first used clinically in 1998 [9] and was subsequently utilized in over half of all THAs within the United States by 2003 [10]. Several studies have demonstrated that first-generation XLPE is associated with a significantly lower annual wear rate compared to UHMWPE as well as a significantly lower rate of radiographic signs of osteolysis [9, 11–13].

Yet despite promising results with XLPE, concerns have been raised regarding how the material will perform in the long-term, specifically with regard to continued oxidation and delamination [14–16]. Furthermore, cobalt chrome femoral heads in metal-on-polyethylene articulations have been implicated in cases of trunnionosis, an increasingly recognized etiology of hip pain and subsequent revision surgery [17, 18]. Therefore, alternative femoral head bearings have been developed to improve wear characteristics. Oxidized zirconium (OxZr, Oxinium, Smith & Nephew, Memphis, TN) is one such example, combining the surface smoothness of a ceramic component with the internal toughness of cobalt chrome. OxZr has been shown to have better wear properties than cobalt chrome and has not been associated with any reported case of trunnionosis [19, 20]. Furthermore, OxZr has been shown to be equivalent to cobalt chrome in the short- to mid-term [21, 22] with regard to linear wear and peri-implant osteolysis. However, no long-term investigations of OxZR with regard to in-vivo polyethylene wear and incidence of osteolysis exist.

The main objective of the current study is to compare the radiographic wear and incidence of osteolysis in the long-term of a cohort of consecutive THA patients that received OxZr femoral heads articulating with either UHMWPE or HCLPE liners. We hypothesized that OxZr on HCLPE would (1) produce a significantly lower annual wear rate and (2) produce a significantly lower incidence of osteolysis compared to UHMWPE. We also hypothesized that OxZr on UHMWPE would produce a long-term annual wear rate below 0.2 mm/yr, which has been previously reported [23, 24] with cobalt chrome on UHMWPE with the same acetabular component utilized in this study.

## Methods

This study received approval from our Institutional Review Board. A retrospective analysis was performed to identify consecutive patients in our institution with minimum 8-year follow-up who received an OxZr-on-polythylene primary THA performed by the senior author (PP) from January 1st, 2003 to December 1st, 2004. The senior author has been utilizing OxZr femoral heads routinely in primary THA since 2003 and the specified follow-up was selected to coincide with the transition from UHMWPE liner to XLPE liner use. Apart from the change in liner composition, all THAs were performed using the posterolateral approach with the same cementless acetabular (Reflection, Smith, & Nephew) and femoral (Synergy, Smith, & Nephew) implants. The most common (>95%) head size utilized at the time was 28 mm and to increase the study’s internal validity, we decided prior to data collection that only THAs performed using a 28-mm head would be included for analysis. Preoperative variables retained for analysis included patient age, gender, and diagnosis. Cup size and head size were recorded from the operative notes. Outpatient notes were reviewed to determine date of most recent follow-up, if revision surgery had been performed surgery and what the indication for revision was. Failure of the bearing surface was defined as the occurrence of revision surgery due to osteolysis or aseptic loosening of the femoral and/or acetabular components.

Radiographic analysis was performed on anteroposterior (AP) pelvis digital X-rays that were taken at the six-week postoperative visit and at the most recent follow-up. X-ray analyses were using the validated Hip Analysis Suite Software (version 8.0.4.5., Martell Hip Analysis Suite™, Chicago, IL) [25–27] and all measures were performed by two experienced observers who were blinded to PE liner type and clinical outcome. The advanced manual mode of the Martell Hip Analysis Software was used to decrease the risk of automated error [28]. Pelvic landmarks, the outline of the femoral head, the center of rotation, and the outline of the acetabular component were manually identified on each AP film. Acetabular component version and inclination were calculated for both initial and final follow-up X-rays, and each AP film’s accompanying shoot-through lateral view was evaluated to assign a positive (anteversion) and negative (retroversion) for version. Annual liner wear rates were calculated through automated comparisons between initial and final follow-up X-rays. Final follow-up X-rays were assessed for evidence of acetabular osteolysis using the DeLee and Charnley method [29].

### Statistical analysis

Data were analyzed using SPSS statistical software (SPSS 23, IBM, NY). For all study variables, a Shapiro–Wilk test was performed to determine the presence or absence of a Gaussian distribution. Patient demographics (age, gender, diagnosis, length of follow-up), implant position (version, inclination), annual liner wear rates, and incidence of osteolysis were compared using Student’s *t*-test, Mann–Whitney *U*-test, or Fisher’s exact test when indicated. Pearson’s correlational coefficient was calculated to determine if any relationship existed between PE wear rates and acetabular position. Inter-rater reliability for radiographic measurements was determined through calculating the intraclass correlation coefficient using a two-way random-effects model assuming a single measurement and absolute agreement. An intra-class correlational coefficient (ICC) of 1 represents perfect reliability, and any value greater than 0.8 indicates excellent reliability [30]. Kaplan–Meier survival curves were plotted according to revision required due to osteolysis or aseptic loosening. For all statistical comparisons, a *p*-value < 0.05 was determined as significant.

A post-hoc power analyses was performed to assess the power of the annual wear measurements between the groups. The post hoc power analysis yielded 100% power to detect a clinically meaningful difference in the annual wear of 0.037 mm with a probability of 95%. This clinically meaningful difference was calculated through averaging the mean annual wear measurements of eight recent long-term HCLPE studies [13, 31–37].

## Results

The retrospective analysis identified 80 patients in the UHMWPE group with an average follow-up of 10.5 ± 1.23 years and 88 patients in the XLPE group with an average follow-up of 10.3 ± 1.03 years (Table 1). Patients in the XLPE group were significantly younger (58.0 ± 10.4 years; *p* < 0.01) compared to the UHMWPE group (64.5 ± 8.67 years). No significant differences were noted in gender (*p* = 0.06) or duration of follow-up (*p* = 0.373). Five patients (6.25%) with UHMWPE liners required revision specifically for aseptic loosening secondary to osteolysis. A sixth UHMWPE patient was revised for a periprosthetic fracture, with acetabular component revision also being performed for an incidental intraoperative finding of severe osteolysis. Conversely, no patients in the XLPE group underwent revision THA for aseptic loosening or osteolysis.

**Table 1:** Patient demographics and radiographic measurements for UHMWPE and XLPE study groups.

	UHMWPE group (mean + SD)	HCLPE group (mean ± SD)	*p*-value
*N*	80	88	–
Age	64.5 + 8.67	58.0 + 10.40	3 × 10^−6^^a^
Gender	47:34	37:49	0.06
Follow-up (years)	10.0 + 1.23	9.9 + 1.10	0.373
Inclination	41.9 + 6.22	42.56 + 6.72	0.322
Ante version	20.5 + 5.57	21.9 + 7.11	0.267
Annual wear rate (mm/year)	0.21 ± 0.12	0.05 + 0.03	1 × 10^−19^^a^
Acetabular osteoly	17 of 80	0 of 88	7.4 × 10^−7^^a^
Zone 1	2	–	–
Zone 2	6	–	–
Zone 3	17	–	–
Femoral osteolysis	37 of 80	0 of 88	9.4 × 10^−15^^a^
Zone 1	21	–	–
Zone 6	2	–	–
Zone7	34	–	–
Other	5	–	–

UHMWPE: ultra-high molecular weight polyethylene, XLPE: highly cross-linked polyethylene, SD: standard deviation.

a Denotes significance of *p* < 0.01.

**Figure 1:**
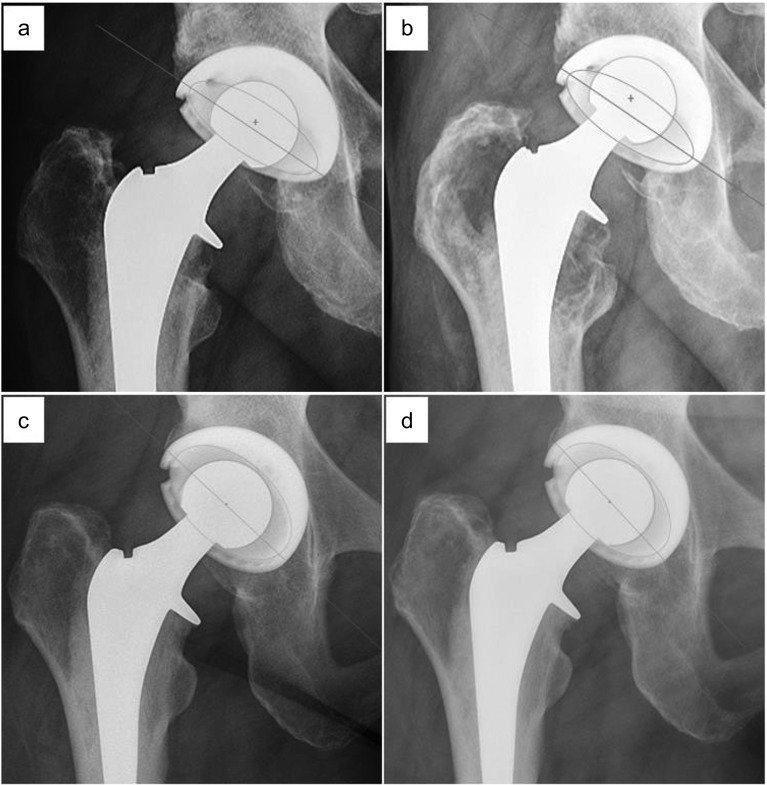
(a) Postoperative and (b) 11.31 year follow-up radiographs of a 28 mm cobalt chrome head on UHMWPE Reflection polyethylene liner. Linear penetration of the head was measured to be 5.581 mm, with observed femoral osteolysis in Gruen zones 1 and 7 (white arrowheads). (c) Postoperative and (d) 10.53 year follow-up radiographs of a 28 mm cobalt chrome head on XLPE Reflection polyethylene liner. Linear penetration of the head was measured to be 0.335 mm, with no observed osteolysis. UHMWPE: ultra-high molecular weight polyethylene, XLPE: highly cross-linked polyethylene.

With regard to radiographic parameters, UHMWPE liners exhibited a significantly higher annual wear rate (0.21 ± 0.12 mm/year) compared to XLPE liners (0.05 ± 0.03 mm/year, *p* = 1 × 10^−19^). These findings were present despite no significant difference existing in implant inclination (*p* = 0.322) or version (*p* = 0.267) between groups. A significant, yet weakly positive correlation was found with increasing annual wear and decreasing acetabular inclination (Pearson’s correlational coefficient: 0.165; *p* = 0.035). Radiographic evidence of osteolysis was noted only in the UHMWPE group (Figure 1). Seventeen (21.2%) UHMWPE patients exhibited acetabular osteolysis at final follow-up, with zone 3 being the most common location (17 of 17) and seven UHMWPE patients demonstrating osteolysis in at least two zones. Thirty-seven (46.3%) UHMWPE patients exhibited femoral osteolysis, with zone 7 being the most common location (24 of 37) and 15 patients demonstrating osteolysis in more than one zone. Inter-observer reliability was excellent for cup abduction (ICC = 0.940), anteversion (ICC = 0.942) and detection of osteolysis (ICC = 0.811).

## Discussion

The long-term success of THA is largely dependent on avoiding periprosthetic osteolysis by minimizing polyethylene wear. In this study, the longest follow-up of oxidized zirconium THA to date, we demonstrated that OxZr coupled with XLPE showed minimal wear and no osteolysis at an average of 10 years following implantation. Conversely, use of UHMWPE was associated with significantly higher rates of wear, osteolysis, and revision for aseptic loosening. Our reported linear wear rate for OxZr on XLPE is similar to a smaller long-term study (0.05 mm/year) [22] and mid-term study (0.061 mm/year) [38], and slightly higher than a mid-term randomized control trial (0.01 mm/year) [21] as well as a mid-term study in younger patients (0.022 mm/year) [39]. As in our own cohort, none of these studies reported any cases of revision THA for osteolysis or aseptic loosening in OxZr-on-XLPE patients.

When compared to other bearing surfaces articulating on XLPE, OxZr remains an appealing choice for long-term THA survival. When compared to ceramic heads, OxZr has demonstrated in clinical follow-up to produce either equivalent [38] or two- to four-fold [21, 22] reductions in annual linear wear rates. Furthermore, OxZr has not been associated with higher rates of tribocorrosion compared to ceramic heads, and in one retrieval study no difference was found in macroscopic fretting and corrosion compared to cobalt chrome [40]. Although a recent hip simulator study [41] has raised concerns for increased wear from OxZR heads that can become scratched following recurrent dislocations, this issue was not observed in the current study due to no episodes of instability.

The strengths of our study include long-term follow-up, multiple reliable assessors for radiographic measurements, and consistent internal validity for surgical technique, head size, and cementless component use. We do acknowledge several limitations in the study. Firstly, the annual wear rate comparison of an immediate postoperative film to most recent follow-up imaging does not uniquely represent true wear (removal of polyethylene) and instead if a combination of true wear with the creep or bedding-in that occurs in the first few months due to non-elastic deformation of the polyethyelene [42]. Secondly, we acknowledge that the computer software utilized for wear analysis is not as sensitive as RSA, widely considered to be the gold standard [43]. Nevertheless, the Martell Hip Analysis software has been widely utilized and in a recent comparative study, was found to be less expensive, less cumbersome, and comparably accurate to both RSA and CT-based wear measurement techniques [44]. Thirdly, although the selected time period for case inclusion was decided upon to maximize follow-up time for the XLPE group, we acknowledge that this may introduce selection bias. Finally, the decision to include only 28-mm OxZr heads, while improving internal validity, also introduces selection bias that could mask possible higher rates of osteolysis that could occur with larger head sizes. Although we acknowledge this limitation for conventional UHMWPE, the effect of head size does not appear to be a significant concern with XLPE [45], with Lachiewicz identifying no relationship between head size and the occurrence of osteolytic lesions in long-term follow-up [46].

The present study reaffirms low wear rates when using XLPE liners in primary THA and provides the longest follow-up of OxZr heads to date. Significantly higher rates of linear wear, radiographic osteolysis, and revision for aseptic loosening were noted with the use of non-crosslinked UHMWPE and consequently, we advise that surgeons consider implementing routine surveillance of patients with these liners once they pass ten years postoperatively. The excellent wear performance of OxZr on the first generation of XLPE liners has positive implications for subsequent XLPE generations, yet similar long-term studies will be needed to verify their equivalence or superiority.
